# Rapid Detection of Dendritic Cell and Monocyte Disorders Using CD4 as a Lineage Marker of the Human Peripheral Blood Antigen-Presenting Cell Compartment

**DOI:** 10.3389/fimmu.2013.00495

**Published:** 2013-12-27

**Authors:** Laura Jardine, Dawn Barge, Ashley Ames-Draycott, Sarah Pagan, Sharon Cookson, Gavin Spickett, Muzlifah Haniffa, Matthew Collin, Venetia Bigley

**Affiliations:** ^1^Human Dendritic Cell Laboratory, Institute of Cellular Medicine, Newcastle University, Newcastle upon Tyne, UK; ^2^Clinical Immunology, Newcastle upon Tyne Hospitals NHS Foundation Trust, Newcastle upon Tyne, UK

**Keywords:** dendritic cells, monocytes, flow cytometry, immunodeficiency, humans

## Abstract

Dendritic cells (DCs) and monocytes are critical regulators and effectors of innate and adaptive immune responses. Monocyte expansion has been described in many pathological states while monocyte and DC deficiency syndromes are relatively recent additions to the catalog of human primary immunodeficiency disorders. Clinically applicable screening tests to diagnose and monitor these conditions are lacking. Conventional strategies for identifying human DCs and monocytes have been based on the use of a lineage gate to exclude lymphocytes, thus preventing simultaneous detection of DCs, monocytes, and lymphocyte subsets. Here we demonstrate that CD4 is a reliable lineage marker for the human peripheral blood antigen-presenting cell compartment that can be used to identify DCs and monocytes in parallel with lymphocytes. Based on this principle, simple modification of a standard lymphocyte phenotyping assay permits simultaneous enumeration of four lymphocyte and five DC/monocyte populations from a single sample. This approach is applicable to clinical samples and facilitates the diagnosis of DC and monocyte disorders in a wide range of clinical settings, including genetic deficiency, neoplasia, and inflammation.

## Introduction

Dendritic cells (DCs) and monocytes are bone marrow derived mononuclear cells involved in a wide range of immune functions. Blood DCs comprise three subsets: plasmacytoid DCs (pDCs), CD1c^+^ myeloid DCs (mDCs), and CD141^+^ mDCs (1–5). pDCs typically lack the myeloid antigens CD13, CD33, and CD11b and express CD123 (IL-3 receptor), CD303 [CLEC4C; Blood DC antigens (BDCA)-2], and CD304 (neuropilin; BDCA-4) (1). They are specialized to produce a rapid type I interferon response to viral infections (6). mDCs share markers in common with monocytes and granulocytes including CD13, CD33, and CD11b and perform the classical functions of DCs in taking up and presenting antigen on HLA class II molecules. DCs resembling all three subsets are found in lymph nodes (7, 8) both mDC subsets have tissue counterparts (5).

Monocytes also comprise a number of distinct functional subsets delineated by expression of CD14 and CD16 in humans. CD14^+^ CD16^−^ “classical monocytes” perform inflammatory functions including phagocytosis, production of reactive oxygen species, nitric oxide, and TNFα (9). Two additional populations have been described: CD16^+^ CD14^low^ “non-classical” monocytes and CD14^+^ CD16^+^ “intermediate” monocytes (3, 10). There is variation in how these cells are divided, with a position paper on nomenclature suggesting that intermediate monocytes may be grouped with non-classical monocytes (both linked by the expression of CD16) while more recent gene expression studies suggest that intermediate monocytes are more closely linked to classical monocytes (11). Both by flow cytometry and gene set enrichment analysis, intermediate monocytes appear to be part of a continuum (12). It is clear, however, that the non-classical pole of the spectrum contains cells with higher class II expression, allo-stimulatory capacity, and cytokine production that have led to their classification as a type of DC (2, 13). CD16^+^ non-classical monocytes are also smaller and become closely associated with the endothelium upon adoptive transfer into mice (11). A wide range of studies show that non-classical monocytes are increased by exercise, autoimmune disease, bacterial sepsis, tuberculosis, and HIV infection, reviewed in Ref. (10).

Routine analysis of human blood DCs and monocytes is usually confined to the enumeration of classical monocytes by automated blood counters. The complexity of changes in DC and monocyte subsets is not visible in most clinical scenarios. Flow cytometry is frequently used to analyze lymphocyte subsets but simultaneous detection of DCs and monocytes is hampered by the lack of a positive lineage marker. Although robust platforms for DC counting have been described, these invariably depend upon identifying MHC class II (HLA-DR) expression by lineage (lin) negative cells, a population defined by the exclusion of T cells (CD3), B cells (CD19, CD20), NK cells (CD56), monocytes (CD14, CD16), and progenitors (CD34) (2, 14, 15). The exclusion of lin^+^ lymphocytes and monocytes either precludes simultaneous measurement with DCs or demands large number of fluorescence channels (4, 15). Differential DC and monocyte counting is therefore rarely performed as a clinical test.

Blood DC antigens 1–4 are helpful for identifying human DC subsets (1) but are often used to define pDC and mDC subsets within the HLA-DR^+^ lin^−^ population (4, 16). This is especially true for CD1c (BDCA-1), which identifies the main population of mDCs but is also expressed on B cells (1). CD303 (BDCA-2; CLEC4C) and CD304 (BDCA-4; neuropilin) are relatively robust markers for pDCs and give reliable counting even from unselected peripheral blood mononuclear cells. CD141 (BDCA-3) defines a very small population of mDCs (1, 2).

Early reports demonstrated that DCs and monocytes both express CD4, but at lower levels than T helper cells (17–19). While the CD4 glycoprotein acts as a co-receptor for the T cell receptor on T helper cells, its role on myeloid cells is less clear. CD4 also functions as a receptor for IL-16 (lymphocyte chemoattractant factor), which is capable of both recruiting CD4^+^ immune cells and driving cell growth (20). Studies in HIV suggest that CD4 may augment Fc receptor signaling (21). Numerous studies report the presence of CD4 on myeloid hematopoietic precursors including myeloid malignancies (22). While in mice, expression of CD4 is retained by T lymphocyte and myeloid precursors but lost by B lymphocyte precursors (23), the function and fate of CD4 expressing precursors in humans has not been adequately mapped.

Here we show that CD4 has significant utility as positive lineage marker of human blood antigen-presenting cells, allowing delineation of all the currently recognized subsets of human blood monocytes and DCs. This allowed us to adapt a standard 6-color flow cytometry protocol for lymphocyte immunophenotyping to allow simultaneous quantification of DC and monocyte subsets.

## Materials and Methods

### Participants

For assay development, blood was collected into EDTA from 33 patients and 5 healthy controls. Patients were randomly selected from the regional clinical immunology laboratory. Patients were aged 2–83 (median 44) and had unknown conditions for further investigation [11], immunodeficiency [7], autoimmunity [7], or atopy [3]. To test the assay in pathological states, fresh blood was obtained from a 33-year old man with DCML deficiency due to GATA-2 deficiency and a 77-year old man with blastic plasmacytoid dendritic cell neoplasm (BPDCN). To recapitulate septic conditions with left-shifted myelopoiesis, eight samples of cryopreserved G-CSF-mobilized peripheral blood stem cells (PBSC) were thawed and washed for analysis. Ethical approval was granted by Newcastle and North Tyneside Research Ethics Committee 1.

### Processing and analysis

TruCount™ tubes (Becton Dickinson; BD) containing a defined number of polyfluorescent beads per test were used in a single step “lyse-no wash” method to enumerate blood cells, according to the manufacturer’s instructions. Fifty microliters of blood was transferred directly to the TruCount™ tube. Antibodies were added directly and staining was performed at 4°C for 20 min. Red cell lysis was achieved by addition of 450 μl of the proprietary reagent at room temperature for 10 min. Samples were then directly analyzed by flow cytometry and the number of cells per microliters of blood was calculated from the number of events and the fraction of beads analyzed, according to the formula:

$$\begin{array}{llll} \mathrm{Cells}∕\mu l\;\mathrm{of}\;\mathrm{population}\;x & =\left(\mathrm{population}\;x\;\mathrm{events∕bead}\;\mathrm{events}\right)\; & & \;\\ & \;\times \left(\mathrm{beads}\;\mathrm{per}\;\mathrm{test}∕50\right)\; & & \; \end{array}$$

The TBNK reagent (BD catalog number 337166) contains six reagents as described in Table 1 for the detection of CD45, CD3, CD4, CD8, CD19, and CD16/56. Combined with TruCount tubes this reagent allows the enumeration of CD4^+^ and CD8^+^ T cells, B cells, and NK cells. The modification for detecting DCs and monocytes was to add CD14-APC-Cy7 to the CD8 channel and CD304-APC to the CD19 channel.

**Table 1:** **Antibody panels used for flow cytometry analysis**.

Fluorochrome	8-Color DC panel	Modified 6-color panel
FITC	CD3 ^a^(345763; BD)	CD3
	CD19 (345776; BD)
	CD20 (345792; BD)	
	CD56 (345811; BD)
PE	CD16 (555407; BD)	CD16, CD56
PERCPCy5.5	CD123 (558714; BD)	CD45
PECy7	CD14 (557742; BD)	CD4
APC	CD141 (130-090-907; Miltenyi)	CD19
		**CD304** (130-090-900; Miltenyi)
APCCy7	CD14 (557742; BD)	CD8
		**CD14** (557831; BD)
V450	CD11c (560369; BD)
V500	HLA-DR (561224; BD)	

*^a^ (Catalog number; supplier)*.

*CD4 V500 (560768; BD), CD4 PE (555347; BD), CD3 PE (345765; BD), CD19 PE (555413; BD), and CD56 PE (345812; BD) were used in additional experiments*.

*Bold text indicates the antibodies added to modify the 6-colour TBNK(TM) panel (337166; BD)*.

Flow cytometry was performed on FACSCanto two or three laser instruments running DiVa version 6 (BD). Instrument PMTs were set using whole blood stained with single antibodies. FSC and SSC thresholds were set at zero to in order to capture TruCount™ bead events. A threshold was set on CD45, allowing detection of leukocytes, but discounting platelet and red cell debris. Thresholds were 1000 on 635 780/60 channel for the 8-color panel and 1000 on 488 760LP channel for the 6-color panel. Isotype controls were performed for all antibodies at the initial set up and are shown specifically for CD4. Automatic compensation was performed using compensation beads (BD). Approximately 200,000 CD45^+^ events were acquired per sample (Table 2). Analysis was performed with FlowJo version 9.5.2 (TreeStar). Graphing and statistical analysis were performed with Prism version 6 (GraphPad).

**Table 2:** **Flow cytometry events recorded for analysis of monocyte and DC subsets**.

Cell population	8-Color DC panel events collected median (IQ range)	Modified 6-color panel events collected median (IQ range)
**DC PANEL COMPARISON (*N* = 24)**		
Total	204634 (125021)	197925 (101726)
CD14 monocytes	24422 (13512)	9289 (5981)
CD16 monocytes	1590 (2973)	570 (934)
CD14 CD16 monocytes	1381 (1443)	633 (499)
DC	971 (697)	369 (327)
**DC SUBSET DISCRIMINATION (*N* = 14)**		
Total	112172 (39906)	130255 (116775)
mDC	408 (218)	239 (98)
pDC	154 (295)	81 (190)

## Results

### CD4 is a lineage marker for blood antigen-presenting cells

**Figure 1:**
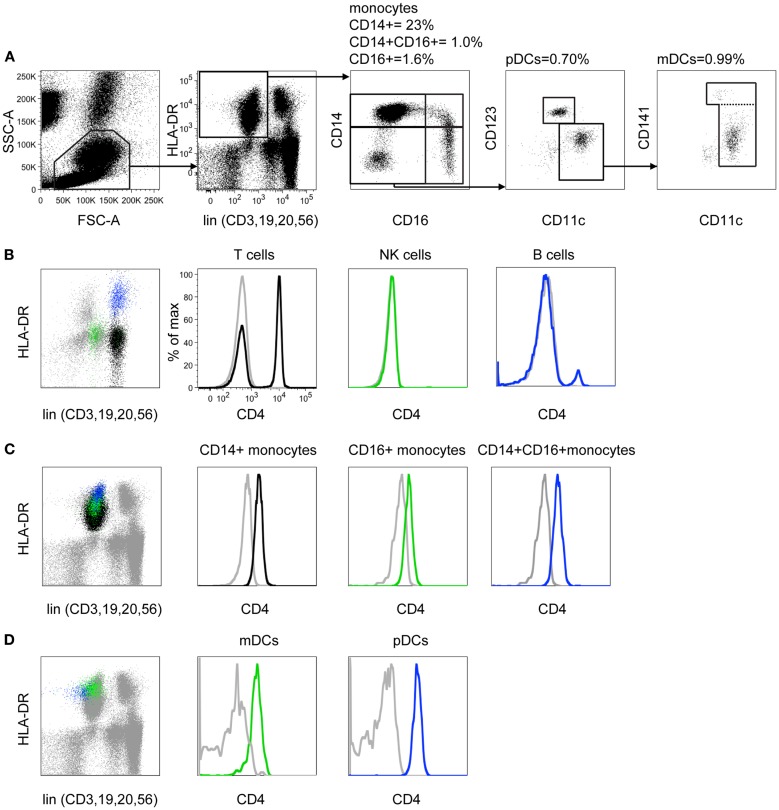
**CD4 is differentially expressed on monocytes and DCs**. **(A)** In an 8-color DC profiling panel FSC and SSC parameters are used to distinguish mononuclear cells from granulocytes, counting beads, and debris. Mononuclear cells are confirmed as CD45^+^ SSC low (gate not shown). Lineage markers (CD3, 19, 20, 56) are used to remove T, B, and NK cells from analysis and DR^+^ monocytes and DCs are selected. The CD14 vs. CD16 plot reveals three subsets of monocytes and CD14^−^ CD16^−^ DCs. Plasmacytoid DCs are defined as CD11c^low^ CD123^+^. Two subsets of CD11c myeloid DC are split by CD141. Percentage values shown indicate the proportion of gated cells relative to CD45^+^ SSC low cells. **(B)** CD3^+^ T cells (black), CD56^+^ NK cells (green), and CD19^+^ B cells (blue) are back-gated onto the lineage vs. DR plot to demonstrate the locations of these populations. Expression of CD4 was then tested (colored histograms) relative to isotype control (gray). Note that a small population of activated DR^+^ CD4^+^ T cells overlaps the B cell population. **(C)** CD14^+^ monocytes (black), CD16^+^ monocytes (green), and CD14^+^16^+^ monocytes (blue) are back-gated onto the lineage vs. DR plot. Expression of CD4 is shown (colored histograms), relative to isotype control (gray). **(D)** Myeloid DC (green) and plasmacytoid DC (blue) are back-gated onto the lineage vs. DR plot. Expression of CD4 is shown (colored histograms), relative to isotype control.

We designed a flow cytometry panel to examine known subsets of monocytes and DCs. Working with whole blood, the standard approach of lineage exclusion and HLA-DR selection required a minimum of eight fluorescence channels to categorize all monocyte and DC subsets. Throughout the study we used this 8-color panel as the standard reference panel for DC and monocyte enumeration (Figure 1A). In the CD45^+^ SSC^low^ gate, we first gated on HLA-DR^+^ lineage negative cells (CD3, CD19, CD20, CD56) to obtain all the monocyte and blood DC populations. Monocytes were analyzed by CD14 vs. CD16 and the double negative cells further separated into pDCs and two subsets of mDCs. We then explored the expression of CD4 on different leukocytes. We used specific fluorophores to identify each lineage-positive fraction and the schema outlined in Figure 1A to identify DCs and monocytes. CD4^+^ T cells are seen in the CD3^+^ fraction but B cells and NK cells are CD4 negative (Figure 1B). Back-gating illustrates where each population lies on the HLA-DR vs. lineage plot. This indicates that NK cells are most likely to broach the lin-DR^+^ gate. As many NK cells express CD16, the gate must be placed sufficiently low on the lineage axis to prevent subsequent NK contamination of the CD16 monocyte gate. All monocyte and DC populations express CD4 above isotype control **(**Figures 1C,D). Back-gating shows the relative HLA-DR expression of these populations. The position of the HLA-DR vs. lineage gate is critical as some monocytes express low HLA-DR. The lower border of the gate on the HLA-DR axis was judged according to isotype controls for HLA-DR (not shown) and the inclusion of a maximum number of CD14^+^ monocytes, the cells with the lowest HLA-DR expression. To ensure that all monocytes and DCs were being captured by this strategy, the gate was also drawn simply as a lineage negative gate (Figure S1 in Supplementary Material). Although this results in the inclusion of HLA-DR negative myeloid precursors and basophils (4), these are subsequently excluded out by the down-stream gating on monocytes and DCs. This at least confirms that no cells are lost by the HLA-DR^+^ lineage negative gate as it was defined in Figure 1A.

### CD4 expression identifies monocytes and DCs within a lymphocyte phenotyping panel

**Figure 2:**
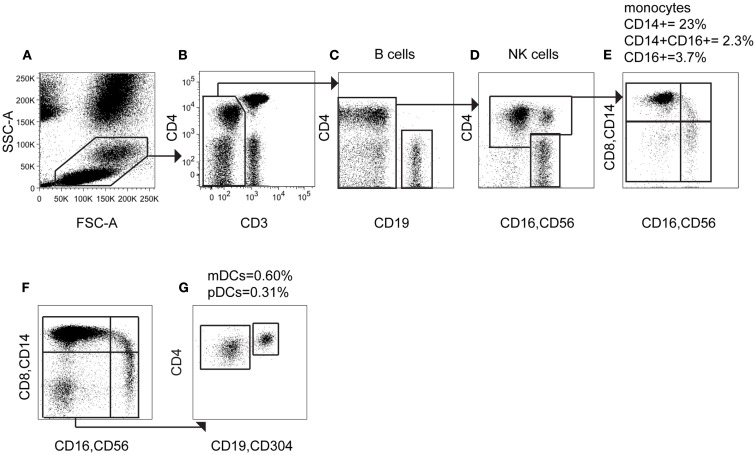
**Using CD4 as a lineage marker allows monocytes and DCs to be counted with a 6-color immunophenotyping panel**. **(A)** In a 6-color immunophenotyping panel modified by addition of CD14 antibody, mononuclear cells are gated based on FSC and SSC parameters. **(B)** CD3 negative cells, including DCs and monocytes, B cells and NK cells are gated out from T cells. **(C)** CD19^+^ B cells are removed from the CD3^−^ population. **(D)** CD16^+^ CD56^+^ NK cells are removed from analysis and the CD4^+^ population selected. **(E)** Visualized on a CD14 vs. CD16 plot, the CD4^+^ population contains three subsets of monocytes and CD14^−^ CD16^−^ DC. **(F)** Adding CD304 antibody to the assay allows further separation of the CD14^−^ CD16^−^ DC population into **(G)** CD4^+^ CD304^−^ mDCs and CD4^bright^ CD304^+^ pDCs.

Having established the expression of CD4 by different leukocytes, we explored the ability of this marker to capture DCs and monocytes. The previous results predicted that a 2D plot of CD4 vs. CD3 should display a population of CD4^+^ CD3^−^ cells containing DCs and monocytes, with slightly lower CD4 expression than CD4^+^ T cells. Both these markers are found within a standard lymphocyte phenotyping panel, offering the potential to enumerate DCs monocytes and lymphocytes simultaneously. We used the TBNK™ reagent (BD) containing six colors to detect T, B, and NK lymphocytes and provide CD4:CD8 ratios from a single tube. As with the previous panel, mononuclear cells are initially separated from granulocytes, beads and debris by FSC and SSC properties (Figure 2A) and CD45^+^ cells selected (not shown). The subsequent 2D plot of CD4 vs. CD3 shows the predicted population of CD4^+^ CD3^−^ cells (Figure 2B) that does not contain B cells (Figure 2C) or NK cells (Figure 2D).

To characterize these cells further, we modified the TBNK reagent by re-using fluorescence channels that had already been excluded by the gating, namely APC (conjugated to CD19) and APCCy7 (conjugated to CD8). CD14 APCCy7 in combination with CD16/56 thus allows resolution of monocyte subsets and the CD14^−^ CD16^−^ parameter space, containing DCs (Figure 2E). Note that the CD4^+^ CD16^+^ population identified in Figure 2D is confirmed as the CD16^+^ non-classical monocyte by low to medium expression of CD14. Further display of the CD14^−^ CD16^−^ population by CD4 vs. CD304-APC enabled the assay to differentiate between mDCs and pDCs (Figures 2F,G). This also confirms that pDCs have the highest CD4 expression among DCs and monocytes. The TBNK reagent and its modification are detailed in Table 1.

### The modified TBNK panel provides accurate enumeration of monocytes and DCs

To provide a proof-of-concept that modification of the 6-color TBNK reagent with CD14 APCCy7 could reproduce the enumeration of DCs and monocytes, we ran 24 samples in parallel with the standard 8-color panel (using HLA-DR vs. lineage as an initial gate). A further 14 samples were run including both CD14 APCCy7 and CD304 APC with the TBNK reagent to evaluate the ability to split DCs into pDCs and mDCs. To reproduce the conditions of a clinical test as closely as possible, we took samples referred to the regional clinical immunology lab and prepared and analyzed them independently on separate machines.

Both panels were run using TruCount tubes to achieve absolute quantification per microliters of blood. Results from the modified TBNK reagent were highly correlated with those of the standard 8-color panel. The least correlated results were produced for total DCs and mDCs. This is probably due to the fact that the 6-color strategy did not include a positive marker of mDCs (except for CD4); they were defined by the exclusion of other populations so it is possible for a variable low level of contaminating cells to be included. Approximately 200,000 total events were recorded in order to collect at least 100 events for rare subsets of DCs (Table 2).

### Detection of DC and monocyte disorders in clinical specimens

**Figure 3:**
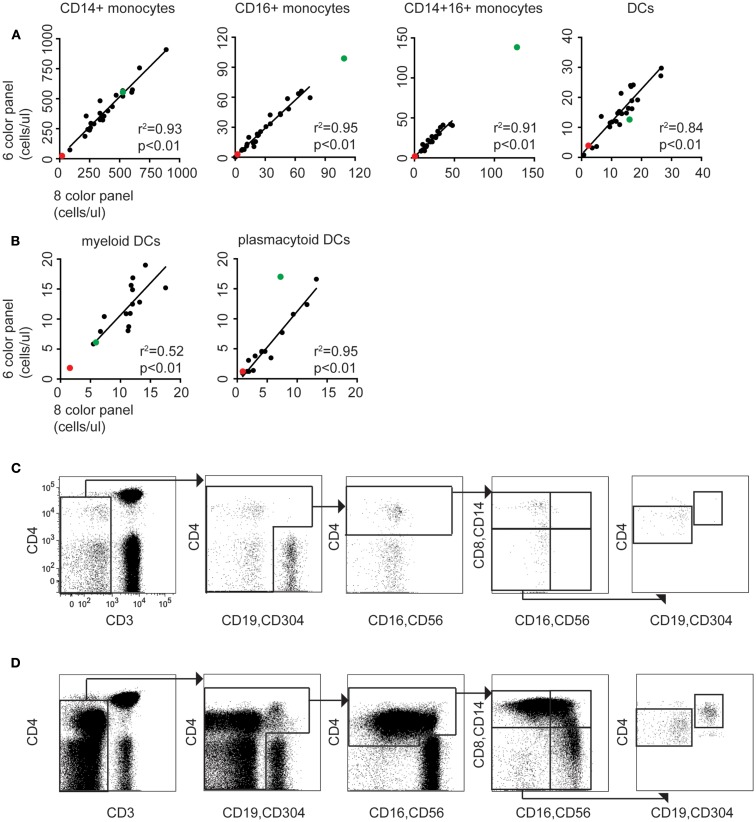
**The modified TBNK assay allows rapid quantitation of APC subsets, consistent with conventional methods**. **(A)** Correlation between monocyte and DC counting performed using the 6-color immunophenotyping panel modified by addition of CD14 vs. the standard 8-color DC panel (*n* = 24). Red dots indicate results for case 1 (DCML deficiency). Green dots indicate case 2 (BPDCN). Linear regressions, *r*^2^ and *p* values stated on the plots exclude these outlier cases. **(B)** Correlation between mDC and pDC counting using the 6-color panel modified with CD14 and CD304 vs. the standard 8-color DC panel (*n* = 14). **(C)** The modified TBNK assay applied to case 1 (DCML deficiency). Note the scarcity of CD3^−^ cells relative to CD3^+^ T cells. This arises from reduction in B cells, absence of NK cells, and virtual absence of all monocyte and DC subsets. The small population of CD3^−^ CD4^bright^ cells gated out in the initial plot are activated T cells with reduced CD3 expression. **(D)** The modified TBNK assay applied to case 2 (BPDCN).

To further test the clinical utility of the modified TBNK reagent, we selected two cases where highly abnormal numbers of DC and monocytes were detected using the 8-color panel and re-examined them with the modified TBNK test. The results are plotted on Figure 3.

#### Case 1

A 33-year-old man presented to rheumatology with a history of pyogenic infections, chronic papillomatosis, and recurrent erythema nodosum. Automated blood counts were normal except for a monocyte count 0.03 × 10^9^/l. DC, monocyte B, and NK lymphoid (DCML) deficiency was suspected and GATA-2 mutation subsequently confirmed by sequencing (24). A blood sample obtained during routine clinical monitoring was analyzed with the modified TBNK panel (Figure 3C). Significant and parallel depletion of all monocyte and DC subsets was observed by both methods.

#### Case 2

A 77-year-old man presented to dermatology with progressive skin nodules and plaques. Immunohistochemistry of a skin biopsy revealed a dense infiltrate of mononuclear cells positive for CD4, CD56, and CD123 consistent with a diagnosis of BPDCN. The leukemic expansion of pDCs was evident with both 8-color and modified TBNK (Figure 3D) tests. In addition, a relative expansion of intermediate and non-classical monocytes was also found by both methods. In the pDC analysis, the 8-color method appeared to underestimate; we attribute this to the expression of CD56 by BPDCN which caused some cells to be lost from the HLA-DR^+^ lineage^−^ gate as they shifted into the lineage+ fraction. In this case, using CD4 as a positive DC marker proved to be a more reliable approach.

### Enumeration of DCs and monocytes in inflammatory states

**Figure 4:**
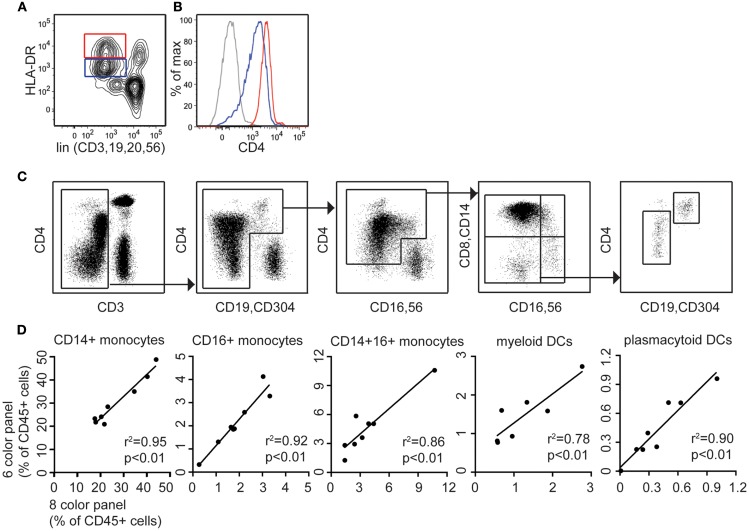
**CD4 is expressed on both DR low and DR bright populations following G-CSF mobilization. The modified TBNK assay allows clear discrimination of lymphocyte, monocyte, and DC populations in left-shifted blood**. **(A)** The lineage DR plot used for 8-color DC profiling shows DR^bright^ and DR^low^ populations of monocytes and DCs in G-CSF mobilized blood. **(B)** CD4 expression is highest on lin^−^ DR^bright^ cells but also positive on lin^−^ DR^low^ cells. **(C)** The modified TBNK assay applied to G-CSF mobilized peripheral blood stem cells (PBSC) permits effective discrimination of lymphocyte, monocyte, and DC populations. **(D)** Correlation between 8-color and modified TBNK methods for DCs and monocytes in PBSC.

Septic shock and other inflammatory conditions are associated with an expansion of monocytes bearing lower HLA-DR. We therefore explored whether detecting monocytes and DCs by CD4 expression would circumvent the problem of counting cells with low HLA-DR expression in an HLA-DR^+^ lineage^−^ gate, or be subject to similar pitfalls. As a model of septic shock we took samples of G-CSF mobilized peripheral blood cells, which also show expanded monocytes with low HLA-DR, and compared counts derived from the two panels. The HLA-DR^low^ fraction of the DC/monocyte gate also expressed lower CD4 (Figures 4A,B) but as with the HLA-DR^+^ lineage^−^ gating approach (Figure S1 in Supplementary Material), it was possible to extend the lower limit of the gate to include the cells with lower expression of CD4 (Figure 4C) resulting in positively correlated results between the two methods (Figure 4D).

## Discussion

In this study we show that CD4 expression may be used within a lymphocyte typing panel to identify DCs and monocytes and that this has clinical utility in enabling the simultaneous detection of nine subsets of lymphocytes, monocytes, and DCs with only six flow cytometry channels.

Dendritic cells and monocytes have been known to express CD4 since the early descriptions of blood DCs and from studies of infectivity by HIV (17–19) but recent strategies for analysis of DCs and monocytes have typically relied upon resolving HLA-DR^+^ lineage^−^ cells (3, 4). CD4 has been used in combination with a lineage cocktail to identify DCs but lymphocytes are still excluded by this approach (16). The use of CD4 as a positive lineage marker, in combination with CD3, preserves the identification of lymphocyte subsets. Multiplexing fluorescence channels with more than one antibody appears to be a reliable means of expanding the number of parameters. Although we cannot exclude that aberrant expression of surface markers in some conditions may distort the analysis, this is true of any flow cytometry assay and our modified 6-color strategy actually enhanced the detection of the malignant CD4^+^ CD56^+^ pDC clone compared with an HLA-DR vs. lineage approach. Employing a single standard mononuclear cell profiling panel and multiplexing antibodies is likely to prove especially beneficial where resources and flow cytometry parameters are restricted, as in many clinical service laboratories. Here we provide proof-of-concept but additional validation and generation of local normal ranges will be required for clinical use.

This type of assay will be useful for identifying DC deficiency as recently described in a number of novel human syndromes (24–26). A typical approach to identifying immunodeficiency uses screening investigations to refine the differential diagnosis followed by more specialist investigations to specify the defect (27). The capacity to enumerate monocytes and DCs as part of an existing lymphocyte immunophenotyping platform, is an important addition to the screening repertoire. Between the 2009 and 2011 revision of the IUIS Primary Immunodeficiency Classification, 7 of the 15 new disease entities concerned the myeloid antigen-presenting cell compartment (28). The development of means to rapidly enumerate DCs and monocytes is therefore an unmet need of clinical importance (29).

Expansion of specific monocyte subsets has been observed in infection and inflammation in many clinical contexts. The delineation of monocytes is not always straightforward in relation to other cells such as NK cells through the expression of CD56 (30) or owing to the loss of HLA-DR expression during neoplasia or inflammation (31, 32). HLA^−^ DR^low^ monocytes also express less CD4. Rigorous comparison of gating strategies has shown that all monocytes may be collected using only HLA-DR, CD14, and CD16 (32); here we show that a CD4 gate with a lower threshold also captures the same events.

Bringing blood DC and monocyte subset analysis into routine clinical practice will most likely reveal even greater variety and subtlety of DC and monocyte deficiency states. New correlations between human disease and the behavior of this compartment are anticipated.

## Author Contributions

Laura Jardine performed experiments, analyzed data, and wrote the manuscript. Dawn Barge designed the study, performed experiments, and analyzed data. Ashley Ames-Draycott performed experiments, Sarah Pagan performed experiments. Sharon Cookson performed experiments. Gavin Spickett commented on the manuscript. Muzlifah Haniffa commented on the manuscript. Matthew Collin designed the study and wrote the manuscript. Venetia Bigley designed the study and wrote the manuscript.

## Conflict of Interest Statement

The authors have no conflicts of interest to disclose. The use and modification of the TBNK™ reagent was by design of the authors and was not influenced in any way by the manufacturer.

## Supplementary Material

The Supplementary Material for this article can be found online at http://www.frontiersin.org/Journal/10.3389/fimmu.2013.00495/abstract
