# Supplementary figures and images for: Rapid Detection of Dendritic Cell and Monocyte Disorders Using CD4 as a Lineage Marker of the Human Peripheral Blood Antigen-Presenting Cell Compartment
Source: Front Immunol. 2013 Dec 27;4:495. doi: 10.3389/fimmu.2013.00495 (PMC3873601; doi:10.3389/fimmu.2013.00495)

# Supplementary Figure 1

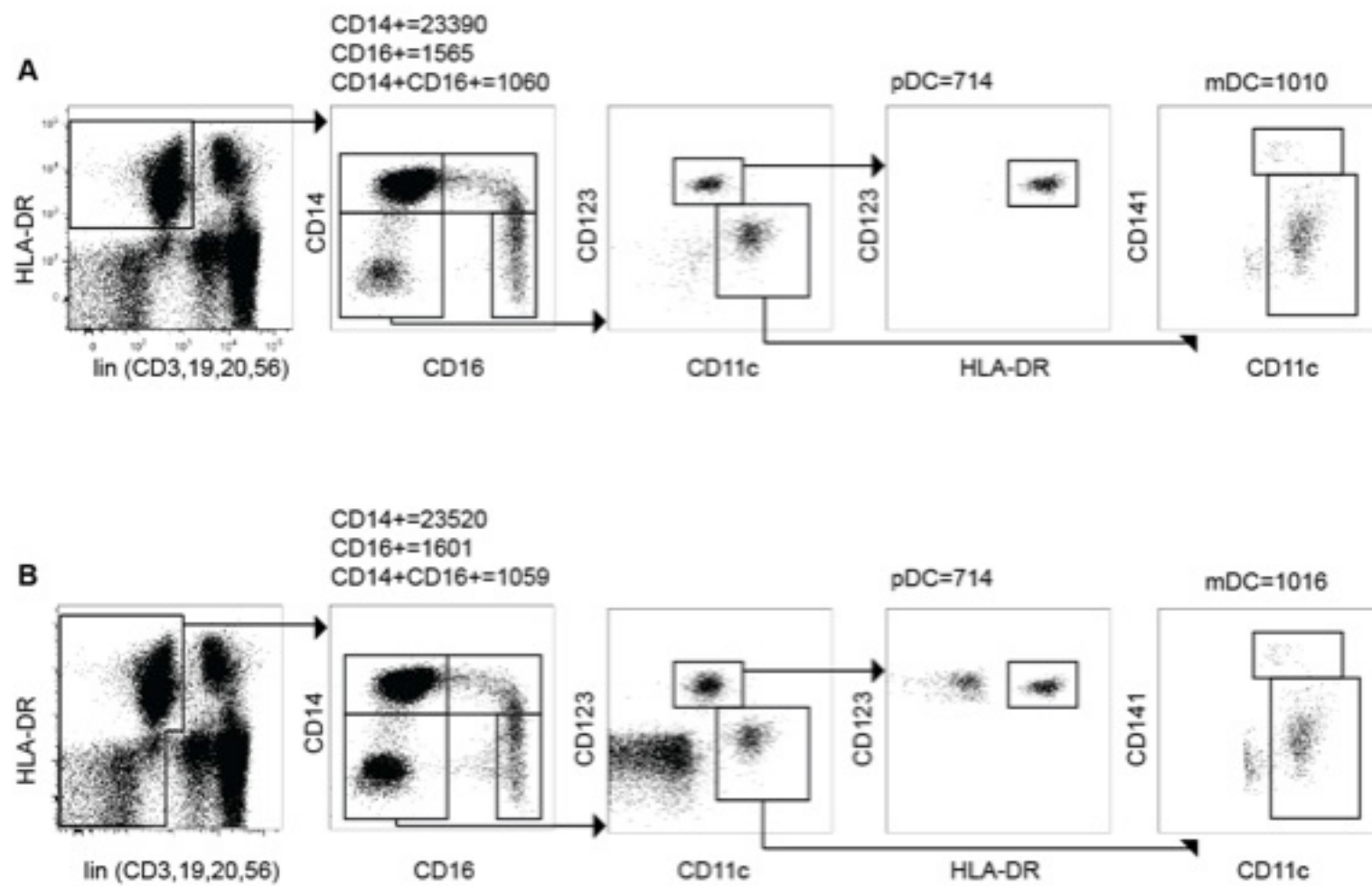

Supplement: Supplementary file 1 [file 71035_Jardine_Presentation1.PDF]
